# Short- and Long-Term Stability of Aromatic Amines in Human Urine

**DOI:** 10.3390/ijerph20054135

**Published:** 2023-02-25

**Authors:** Shrila Mazumder, Rayaj A. Ahamed, Tiffany H. Seyler, Lanqing Wang

**Affiliations:** Tobacco and Volatiles Branch, Division of Laboratory Sciences, National Center for Environmental Health, Centers for Disease Control and Prevention, Atlanta, GA 30341, USA

**Keywords:** aromatic amines, short-term stability, long-term stability, human urine

## Abstract

Several aromatic amines (AAs) are established by the International Agency for Research on Cancer as carcinogenic (group 1) or probable/possible carcinogens to humans (group 2A/2B). AAs can be found in mainstream and sidestream smoke from combustible tobacco products, as well as in certain environmental pollution and occupational exposure from several chemical industry sectors. Exposure to AAs can be estimated by measuring their concentrations in urine; however, information about the short-term and long-term stabilities of AAs in urine need to be characterized before conducting large-scale population studies on AA exposure and the potentially harmful effects of AA exposure. In this report, the storage stability of o-toluidine, 2,6-dimethylaniline, o-anisidine, 1-aminonaphthalene, 2-aminonaphthalene, and 4-aminobiphenyl fortified in pooled, filtered, non-smokers’ urine is analyzed by isotope dilution gas chromatography-triple quadrupole mass spectrometry (ID GC-MS/MS). The six AAs were measured in urine samples stored at ~20 °C (collection temperature), 4 °C and 10 °C (short-term transit temperatures), and −20 °C and −70 °C (long-term storage temperatures) over a 10-day period. All six analytes were stable for 10 days at transit and long-term storage temperatures but showed reduced recovery at 20 °C. The instability of the target AAs at 20 °C suggests that immediate storage of freshly voided urine at low temperatures is needed to attenuate degradation. A subset of the urine samples was analyzed following a longer storage duration at −70 °C: all AAs were stable for up to 14 months at this temperature. The stability of the six AAs in urine samples can be maintained at the various temperature levels and storage times expected in a typical study set.

## 1. Introduction

Research over the last 70+ years has linked cigarette smoking to numerous health conditions, including an increased risk of cancer, heart disease, and respiratory illnesses, among adults and children [1,2,3,4,5,6,7]. The smoke from combusted tobacco products may be a major source of exposure to aromatic amines (AA) [8,9,10], which have been proposed to be the leading agents for bladder cancer in humans [11]. Furthermore, occupational exposure to AAs may exist from the dyes and pigments industry, pharmaceuticals, pesticides, herbicides, plastics, and synthetic rubber [12,13,14,15,16]. Environmental pollution, such as diesel exhaust, wood and rubber combustion, emissions from cooking oils, and combustion products from charcoal-barbequed meats, may also contribute to exposure [17,18,19]. Several AAs have been classified as carcinogenic (group 1) or probable/possible carcinogenic to humans (group 2A/2B) by the International Agency for Research on Cancer [11,20,21], whereas some also appeared on the Food and Drug Administration’s list of harmful and potentially harmful constituents since the enactment of The Family Smoking Prevention and Tobacco Control Act in 2009 [22]. 

**Figure 1:**
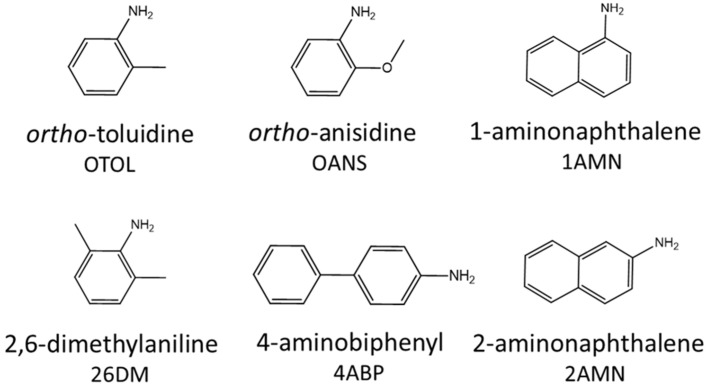
Structures of six aromatic amines assayed for stability testing.

Exposure to AAs can be estimated by measuring the concentration of AAs in urine [23,24,25,26,27,28]. In order to ensure the validity of AA measurements in large studies such as the National Health and Nutrition Examination Survey (NHANES), the stability of urinary AA concentrations must be analyzed and documented. In this report, we monitored both the short-term (up to 10 days) and long-term (over 14 months) stability of all six AAs in spiked human urine. The six AAs monitored in this study are o-toluidine (OTOL), 2,6-dimethylaniline (26DM), o-anisidine (OANS), 1-aminonaphthalene (1AMN), 2-aminonaphthalene (2AMN), and 4-aminobiphenyl (4ABP) (Figure 1). The short-term stability study covered a range of temperatures at which samples are typically stored during the collection and transport stages: samples are collected at room temperature (approximately 20 °C) and transported in chilled shippers ranging from 4 °C to 10 °C. Once delivered to the destination laboratory, samples are typically kept in −20 °C freezers for short-term storage or −70 °C freezers for long-term storage. For the long-term stability study, the samples were stored only at −70 °C and analyzed over a time frame that is sufficient to encompass or exceed the storage time anticipated for a typical study. The results from this report can aid in designing and validating procedures for the collection, transportation, and storage of urine specimens for subsequent AA analysis.

## 2. Materials and Methods

### 2.1. Materials

Native, or un-labeled, compounds used to make the calibration standards were purchased from Fluka, Aldrich, and Sigma (St. Louis, MO, USA). OTOL-13C6, OANS-2H7, and 1AMN-2H9 were purchased from Medical Isotope (Pelhem, NH, USA); 2AMN-2H7 was purchased from CDN Isotopes (Pointe-Claire, QC, Canada); 4ABP-2H9 was purchased from Cambridge Isotope Laboratory (Andover, MA, USA); and 26DM-2H6 was purchased from Toronto Research Chemical (North York, ON, Canada). Semiconductor-grade sodium hydroxide (NaOH) pellets were purchased from Sigma. High-purity hydrochloric acid (HCl), pentafluoropropionic anhydride (PFPA), and trimethylamine hydrochloride (TMA-HCl) were purchased from Sigma Aldrich. All solvents and water were purchased from Burdick and Jackson Labs, distributed by VWR (Suwanee, GA, USA); all ultra-high purity gases were supplied by Airgas (Chamblee, GA, USA). Isolute™ SLE cartridges were purchased from Biotage (Charlotte, NC, USA); 4.5-mL high recovery sample vials were purchased from ChemGlass (Vineland, NJ, USA); EP Scientific 10-mL silanized glass tubes were purchased from LabDepot (Dawsonville, GA, USA); and Wheaton 1-mL amber vials with a 300-µL insert and SUN-Sri 11-mm aluminum crimp caps with rubber septum were purchased from ThermoFisher Scientific (Suwanee, GA, USA). Parts and consumables for the gas chromatograph (GC) and mass spectrometer (MS) were purchased from Agilent Technologies (Santa Clara, CA, USA). Filter tips for sample aliquoting (and any other parts and consumables) in the Hamilton Microlab STAR™ Liquid Handling Workstation were purchased from the Hamilton Company (Reno, NV, USA).

### 2.2. Quality Control Pools

Two quality control (QC) urine pools were created from filtered, in-house donations of non-smokers’ urine by spiking at a low (QC Low) and a high level (QC High) with the six AAs. The collection of urine samples is based on Institutional Review Board protocol 3994. A subset of each of the QC pools (to be used for stability testing) was aliquoted and stored at the following temperatures—18 samples each at −20 °C, 4 °C, 10 °C, and room temperature (approximately 20 °C)—and the remaining aliquots were stored at −70 °C. Ninety-two replicates of the QC Low and QC High samples, stored at −70 °C, were analyzed for 19 months and evaluated (characterized) according to the modified Westgard rules [29]. QC characterization statistics (characterized mean ± 2σ from mean) were subsequently used to verify measurement of each QC pool analyzed in this study.

### 2.3. Automated Sample Preparation and GC-MS/MS Analysis

Samples stored at the five temperature levels were prepared and run in a single analytical batch. The “total” (conjugated and “free” forms) concentration of six AAs is quantified by an isotope-dilution gas chromatographic, tandem mass spectrometric method (ID GC-MS/MS), as reported by Mazumder et al. in a previous publication [30]. Briefly, urine samples were collected and stored at approximately −70 ± 10 °C before further analysis. 13C- and 2H-labeled internal standards were added to the urine aliquots, after which the conjugated analytes were basic hydrolyzed, then cleaned up and extracted on supported liquid extraction (SLE) cartridges. All target analytes were derivatized to form pentafluoropropionamides, reconstituted in toluene, and analyzed by GC-MS/MS using multiple reaction monitoring (MRM). Supplemental Figure S16 shows the total ion counts (TIC) of the six quantified AAs and 11 structural isomers to validate isomeric separation from the target analytes. We monitored one quantitation transition, one confirmation transition, and one corresponding internal standard transition for each analyte quantified. The analyte concentrations were derived from the ratio of the integrated peaks of native to labeled ions by comparing to a standard curve.

## 3. Results

**Figure 2:**
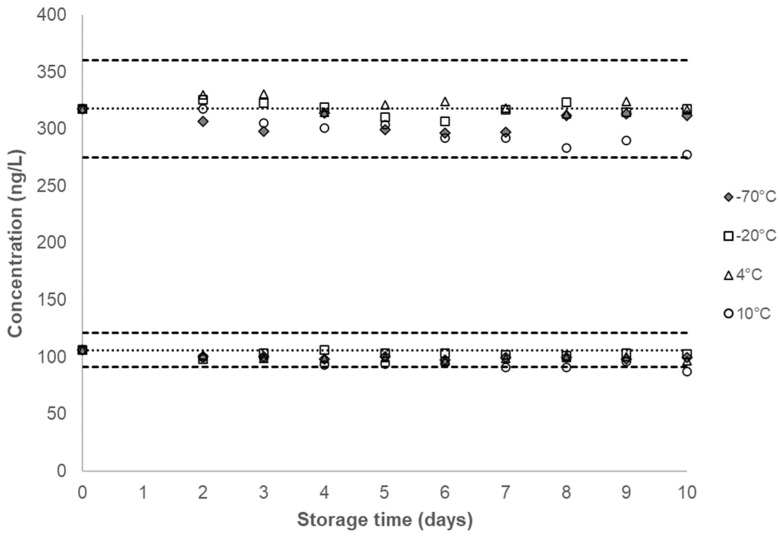
Single time-point urinary concentration of 4ABP stored at the different storage conditions for short-term stability testing. Dotted line denotes the characterized mean for a given QC pool and corresponding dashed lines denote ±2σ from the characterized mean, as listed in Table 1. The characterized mean for a given QC pool is included as the zero time-point.

**Figure 3:**
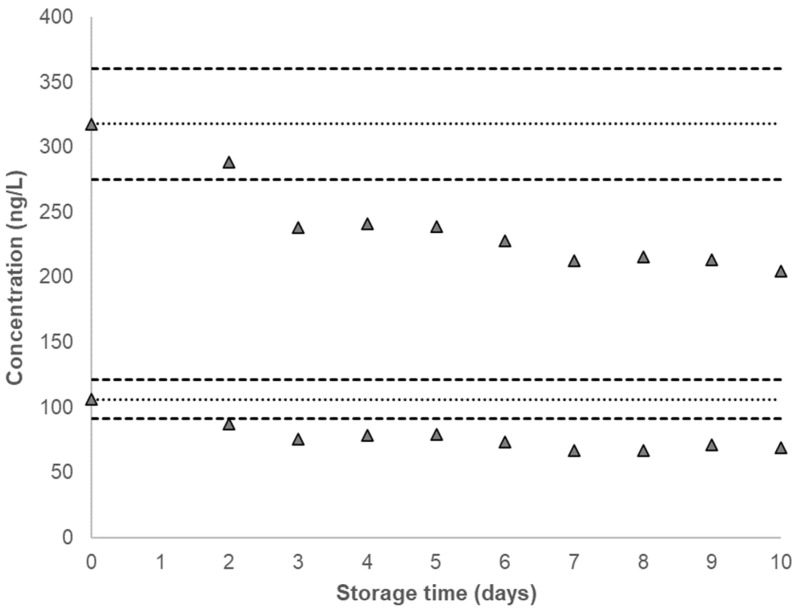
Single time-point urinary concentration of 4ABP stored at 20 °C for short-term stability testing. Dotted line denotes the characterized mean for a given QC pool and corresponding dashed lines denote ±2σ from the characterized mean, as listed in Table 1. The characterized mean for a given QC pool is included as the zero time-point.

**Figure 4:**
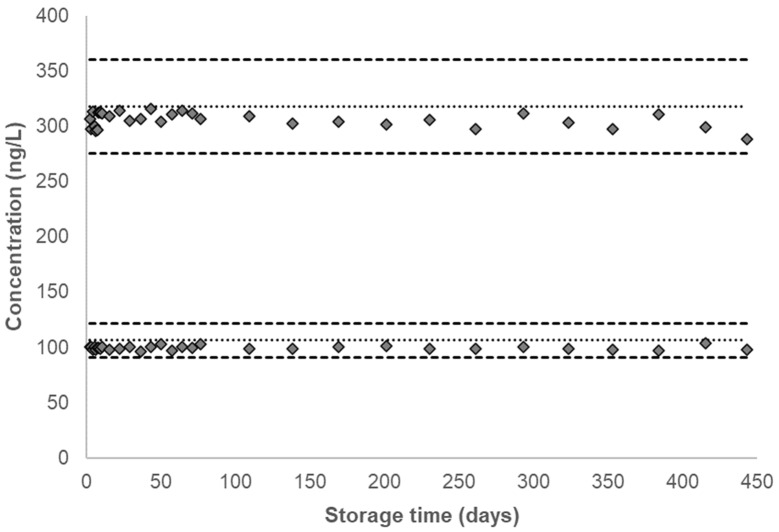
Single time-point urinary concentration of 4ABP stored at −70 °C for long-term stability testing. Dotted line denotes the characterized mean for a given QC pool and corresponding dashed lines denote ±2σ from the characterized mean, as listed in Table 1. The characterized mean for a given QC pool is included as the zero time-point.

Over the duration of the characterization period or 19 months after the QC pools were prepared, no significant changes in concentration were observed for any analyte in either QC pool. For each analyte, the calculated concentrations from both QC pools were plotted after overlaying the corresponding mean analyte concentration along with the ±2σ interval (Figure 2, Figure 3 and Figure 4, Supplemental Figures S1–S15). Table 1 provides the QC characterization statistics and the summary statistics of each QC pool at the short-term and long-term storage conditions. The effect of repeated thaw (at approximately 20 °C) and re-freeze (at −70 °C) cycles on unprocessed urine samples and the effect of provisional storage of processed urine samples at −20 °C were determined for each analyte in a previous study [30]. The results indicated analyte stability following at least five thaw and re-freeze cycles and up to 13 weeks of storage stability of AAs in processed urine samples stored at −20 °C.

### 3.1. Short-Term Stability of Analytes in Human Urine at Various Temperatures

No marked decrease in concentration was observed for any of the analytes in either QC pool at −20 °C, 4 °C, 10 °C and −70 °C over a 10-day period (Figure 2, Supplemental Figures S1–S5). Our study showed a substantial decrease in the measured concentration in most analytes, for both QC pools stored at 20 °C, after two days (Figure 3, Supplemental Figures S11–S15). When stored at −70 °C, −20 °C, 4 °C, and 10 °C, the coefficients of variation (CVs) for all analytes in both QC pools are below 10%, with CVs for all analytes in the QC High recorded to be at or below 6% (Table 1).

### 3.2. Long-Term Stability of Analytes in Human Urine at −70 °C

To test the long-term storage stability, QC pools stored at −70 °C were analyzed past the duration of the short-term stability experiment, up to 443 days, with a total of 31 data points obtained over this period. No marked decrease in concentration was observed for any analyte (Figure 4, Supplemental Figures S6–S10). As shown in Table 1, the CVs for all analytes in both pools are below 6%. At −70 °C, the long-term stability of all analytes is comparable to the degree of stability recorded at the short-term storage conditions.

## 4. Discussion

Samples in large studies are usually collected over several months, shipped at variable temperatures, and potentially stored for years before and after analysis. Therefore, the stability of the analytes must be maintained in order to validate any generated data. The short-term stability results show that measurement of AAs (OTOL, 26DM, OANS, 1AMN, 2AMN, and 4ABP) remain stable under various storage conditions, from typical transit (4 °C to 10 °C) to destination facility (−20 °C to −70 °C), for up to 10 days. This 10-day period allows for samples collected in remote locations to be shipped back to the destination laboratories for analyses, given that the samples are refrigerated (at up to 10 °C) as soon as possible to minimize analyte degradation. In addition, measurement of these six AAs in human urine was stable for at least up to one year if samples were frozen at −70 °C or lower. A different set of QC pools were used to analyze AAs in urine samples from the NHANES 2013–2014 cycle over a period of approximately 36 months: under long-term storage conditions at −70 °C, stability can be achieved for up to 36 months, if needed. This time frame is sufficient to encompass or exceed the storage time anticipated to analyze samples in a typical study set. Furthermore, the relatively similar precisions recorded for both the high and low QC pools (within 5% CVs for most analytes) reflect negligible variance changes across the two concentration regimes analyzed. 

AAs are not stable in urine at 20° C, and measured concentrations decline rapidly after two days of storage, which suggests that analysis of these compounds may not be valid when immediate storage of freshly voided urine at low temperatures is not possible. A review of the literature yields four storage stability studies that were conducted alongside regular method validation tests, covering some of the AAs in our assay panel [31,32,33,34]. The study by Holland et al. [31] tested the 20 °C storage stability of ABP isomers, including 4ABP, for up to six days. This study found ABPs to not be stable at 20 °C, where only 25% of the spiked amount was measured after the sixth day of storage. Riedel et al. [32] tested 20 °C stability of urinary OTOL, 2AMN, and 4ABP for only up to two days; two other studies [33,34] tested 20 °C stability of benzidine and its metabolites, but neither one of these studies exceeded 65 h (>2.5 days) of testing. These studies indicated some degradation of “free” (unconjugated) AAs spiked in urine at 20 °C. pH-adjustment to acidic conditions was shown to improve room temperature storage stability for one of the benzidine metabolites and both ABP isomers, but none of the other studies had carried out similar tests. Furthermore, we can also note some differences in the extent of AA stability at 20°C storage. Although all AAs in this assay panel are known to be air- and light-sensitive, any differences in their sensitivity levels (potentially owing to differences in their chemical structures) may be responsible for such observation. 

Our experimental design of preparing and analyzing all QC pools, from various temperature levels and time points, in one batch, is intended to eliminate or minimize the sources of between-run variation (e.g., calibration error, variances in analytical column quality, and reagents used for sample preparation, etc.) on the calculated concentrations of the QC pools and on the precision of the stability estimates. With this approach, we have a higher confidence in attributing the recorded variability between individual measurements due to the inherent thermal (or other) stability factors of the analyte. 

Extending on the previous point, we note that this study was designed to test the thermal stability of the target AAs and not other factors such as light irradiation and bacterial degradation. Many AAs possess the property of discoloration in the presence of artificial light or sunlight [35,36,37]. Photo-decomposition mechanisms are different depending on the type/class of AA and external conditions [36,37,38], however, these studies may suggest the chemically labile property of some AAs upon irradiation. To minimize the potential effects of irradiation on AAs, we store our reagents in amber vials, in freezers, away from light exposure. Biodegradation of monocyclic AAs has also been studied in a variety of bacteria, where several degradation pathways have been proposed and related enzymes characterized [39]. In this stability study, we have precluded the effects of microbial activity on AA measurements by filtering our QC pools using 0.2 µm filters and storing them at or below −70 °C. However, as urine samples from actual study participants are not filtered after collection and before refrigeration (specifically, at 4 °C or 10 °C), there may be some opportunity for AA biodegradation within such samples. 

Our current study tested the thermal stability of “free” AAs in urine but not the conjugated (glucuronidated and acetylated) forms. A previous method cross-validation test, completed using a “historical” QC pool (prepared in 2006) containing conjugated forms of the AAs, allowed us to verify that the amount of conjugated 2AMN, 4ABP, and OTOL in human urine had not changed over a period of approximately 11 years after continuous storage at −70 °C. However, as original characterization statistics for only 2AMN, 4ABP, and OTOL were established for the historical QC pool, we are unable to verify the long-term stability of the conjugated forms of 1AMN, 26DM, and OANS. Furthermore, we have not tested the short-term storage stability of any of the conjugated AAs at 20 °C. The literature, to our knowledge, does not contain any studies on the room temperature stability of the conjugated AAs, suggesting that the 20 °C stability of the conjugated forms is not well-known. The concentrations of the conjugated forms of the AAs are present in varying amounts in human urine and usually exceed that of the “free” forms. As such, testing the stability of the conjugated forms would be equally relevant in determining the maximum-allowed storage temperature of urine samples reserved for AA measurements.

## 5. Conclusions

Data obtained in this stability study demonstrate that the six target AAs are stable in urine samples for all the storage temperature levels and time points tested, except at room temperature (~20 °C). According to these results, urine samples can be stored at up to 10 °C, for at least 10 days, without incurring substantial analyte loss. Furthermore, our current study and previous analyses have shown that adequate long-term storage of urine samples at −70 °C can be achieved for at least 14 months and up to 36 months.

## Figures and Tables

**Table 1:** Summary statistics and characterization statistics obtained in the quantification of urinary aromatic amines, for two QC pools, from up to 10 days of storage at −70 °C, −20 °C, 4 °C and 10 °C and from up to 443 days of storage at −70 °C.

		QC Low		QC High	
**Study Type**	**Analyte**	**Mean ± 2σ (** **ng/L) (^a^ *n* = 36)**	**CV (%)**	**Mean ± 2σ (** **ng/L) (^a^ *n* = 36)**	**CV (%)**
Short-term stability (−70 °C, −20 °C, 4 °C, 10 °C)	1AMN	92.0 ± 9.6	5.23	272.7 ± 32.5	5.96
	2AMN	82.4 ± 8.5	5.16	253.5 ± 23.7	4.67
	26DM	143.2 ± 9.2	3.21	411.9 ± 27.1	3.29
	4ABP	98.7 ± 7.7	3.92	309.8 ± 26.1	4.21
	OANS	133.8 ± 11.8	4.42	359.2 ± 21.3	2.96
	OTOL	181.0 ± 29.8	8.25	429.5 ± 53.6	6.24
		**Mean ** **± 2** **σ (ng/L) (^b^ *n* = 31)**	**CV (%)**	**Mean ** **± 2** **σ (ng/L) (^b^ *n* = 31)**	**CV (%)**
Long-term stability (−70 °C)	1AMN	97.0 ± 5.2	2.69	280.9 ± 16.3	2.91
	2AMN	86.2 ± 3.6	2.12	259.9 ± 12.9	2.49
	26DM	138.8 ± 8.6	3.11	406.7 ± 43.9	5.39
	4ABP	99.4 ± 3.4	1.69	305.7 ± 13.4	2.19
	OANS	132.9 ± 8.4	3.14	362.3 ± 18.4	2.55
	OTOL	171.4 ± 20.2	5.91	409.8 ± 42.5	5.18
		**Mean ** **± 2** **σ (ng/L) (^c^ *n* = 92)**	**CV (%)**	**Mean ** **± 2** **σ (ng/L) (^c^ *n* = 92)**	**CV (%)**
QC characterization	1AMN	100.6 ± 12.6	6.24	292.6 ± 36.3	6.20
	2AMN	92.0 ± 12.9	6.99	283.5 ± 33.6	5.93
	26DM	148.4 ± 18.7	6.31	430.2 ± 46.3	5.38
	4ABP	106.2 ± 15.1	7.11	317.8 ± 42.6	6.70
	OANS	140.8 ± 15.8	5.59	387.2 ± 35.1	4.54
	OTOL	185.6 ± 67.0	18.05	445.9 ± 85.4	9.57

^a^: one measurement per day, for nine days, at four temperatures (−70 °C, −20 °C, 4 °C and 10 °C); ^b^: one measurement per day, for nine days, and one measurement per week, for 22 weeks, at −70 °C; ^c^: characterization was completed using individual measurements from 92 replicates.

## Data Availability

The data presented in this study are available upon request from the corresponding author.

## Supplementary Materials

The following supporting information can be downloaded at: https://www.mdpi.com/article/10.3390/ijerph20054135/s1, Figure S1: Single time-point urinary concentration of 1AMN stored at the different storage conditions for short-term stability testing; Figure S2: Single time-point urinary concentration of 2AMN stored at the different storage conditions for short-term stability testing; Figure S3: Single time-point urinary concentration of 26DM stored at the different storage conditions for short-term stability testing; Figure S4: Single time-point urinary concentration of OANS stored at the different storage conditions for short-term stability testing; Figure S5: Single time-point urinary concentration of OTOL stored at the different storage conditions for short-term stability testing; Figure S6: Single time-point urinary concentration of 1AMN stored at −70 °C for long-term stability testing; Figure S7: Single time-point urinary concentration of 2AMN stored at −70 °C for long-term stability testing; Figure S8: Single time-point urinary concentration of 26DM stored at −70 °C for long-term stability testing; Figure S9: Single time-point urinary concentration of OANS stored at −70 °C for long-term stability testing; Figure S10: Single time-point urinary concentration of OTOL stored at −70 °C for long-term stability testing; Figure S11: Single time-point urinary concentration of 1AMN stored at 20 °C for short-term stability testing; Figure S12: Single time-point urinary concentration of 2AMN stored at 20 °C for short-term stability testing; Figure S13: Single time-point urinary concentration of 26DM stored at 20 °C for short-term stability testing; Figure S14: Single time-point urinary concentration of OANS stored at 20 °C for short-term stability testing; Figure S15: Single time-point urinary concentration of OTOL stored at 20 °C for short-term stability testing.
