# Supplementary material for: Short- and Long-Term Stability of Aromatic Amines in Human Urine
Source: Int J Environ Res Public Health. 2023 Feb 25;20(5):4135. doi: 10.3390/ijerph20054135 (PMC10002391; doi:10.3390/ijerph20054135)

Short and Long-term Stability of Aromatic Amines in Human Urine

Shrila Mazumder[1], Rayaj A. Ahamed[1], Tiffany H. Seyler[1], and Lanqing Wang[1]

[1] Tobacco and Volatiles Branch, Division of Laboratory Sciences, National Center for Environmental Health, Centers for Disease Control and Prevention, Atlanta, Georgia

Corresponding author:
Tiffany H. Seyler, (770) 488-4527 (Phone), (404) 638-5314 (FAX), E-mail: TSeyler2@cdc.gov

** The findings and conclusions in this study are those of the authors and do not necessarily represent the views of the U.S. Department of Health and Human Services, or the U.S. Centers for Disease Control and Prevention. Use of trade names and commercial sources is for identification only and does not constitute endorsement by the U.S. Department of Health and Human Services, or the U.S. Centers for Disease Control and Prevention.*

Keywords: aromatic amines, short-term and long-term stability, human urine

Supplemental figures = 16

Abstract

Several aromatic amines (AAs) are established by the International Agency for Research on Cancer as carcinogenic (group 1) or probable/possible carcinogens to humans (group 2A/2B). AAs can be found in mainstream and sidestream smoke from combustible tobacco products, as well as in certain environmental pollution and occupational exposure from several chemical industry sectors. Ex-posure to AAs can be estimated by measuring their concentrations in urine, however, information about the short-term and long-term stabilities of AAs in urine need to be characterized before conducting large-scale population studies on AA exposure and the potentially harmful effects of AA exposure. In this report, the storage stability of o-toluidine, 2,6-dimethylaniline, o-anisidine, 1-aminonaphthalene, 2-aminonaphthalene, and 4-aminobiphenyl fortified in pooled, filtered, non-smokers' urine is analyzed by isotope dilution gas chromatography-triple quadrupole mass spectrometry (ID GC-MS/MS). The six AAs were measured in urine samples stored at ~20°C (col-lection temperature), 4°C and 10°C (short-term transit temperatures), and -20°C and -70°C (long-term storage temperatures) over a 10-day period. All six analytes were stable for 10 days at transit and long-term storage temperatures but showed reduced recovery at 20°C. The instability of the target AAs at 20°C suggests that immediate storage of freshly voided urine at low temperatures is needed to attenuate degradation. A subset of the urine samples was analyzed following a longer storage duration at -70°C: all AAs were stable for up to 14 months at this temperature. The stability of the six AAs in urine samples can be maintained at the various temperature levels and storage times expected in a typical study set.

Supplemental Figures

Supplemental Figure S1 – Single time-point urinary concentration of 1AMN stored at the different storage conditions for short-term stability testing
Supplemental Figure S2 – Single time-point urinary concentration of 2AMN stored at the different storage conditions for short-term stability testing
Supplemental Figure S3 – Single time-point urinary concentration of 26DM stored at the different storage conditions for short-term stability testing
Supplemental Figure S4 – Single time-point urinary concentration of OANS stored at the different storage conditions for short-term stability testing
Supplemental Figure S5 – Single time-point urinary concentration of OTOL stored at the different storage conditions for short-term stability testing
Supplemental Figure S6 – Single time-point urinary concentration of 1AMN stored at -70°C for long-term stability testing
Supplemental Figure S7 – Single time-point urinary concentration of 2AMN stored at -70°C for long-term stability testing
Supplemental Figure S8 – Single time-point urinary concentration of 26DM stored at -70°C for long-term stability testing
Supplemental Figure S9 – Single time-point urinary concentration of OANS stored at -70°C for long-term stability testing
Supplemental Figure S10 – Single time-point urinary concentration of OTOL stored at -70°C for long-term stability testing
Supplemental Figure S11 – Single time-point urinary concentration of 1AMN stored at 20°C for short-term stability testing
Supplemental Figure S12 – Single time-point urinary concentration of 2AMN stored at 20°C for short-term stability testing
Supplemental Figure S13 – Single time-point urinary concentration of 26DM stored at 20°C for short-term stability testing
Supplemental Figure S14 – Single time-point urinary concentration of OANS stored at 20°C for short-term stability testing
Supplemental Figure S15 – Single time-point urinary concentration of OTOL stored at 20°C for short-term stability testing
Supplemental Figure S16 – The TIC of six quantified AAs and two of the monitored isomers (2ABP and 3ABP) in an urine sample containing approximately 100 ng/L of each target analyte (represented as dashed line) is overlaid on the TIC of a calibration standard containing 100 pg/µL of each target analyte (represented as solid line). The three insets show the related structural isomers of OANS, OTOL and 26DM, each spiked at 100 pg/µL.

Supplemental Figure S1: Single time-point urinary concentration of 1AMN stored at the different storage conditions for short-term stability testing

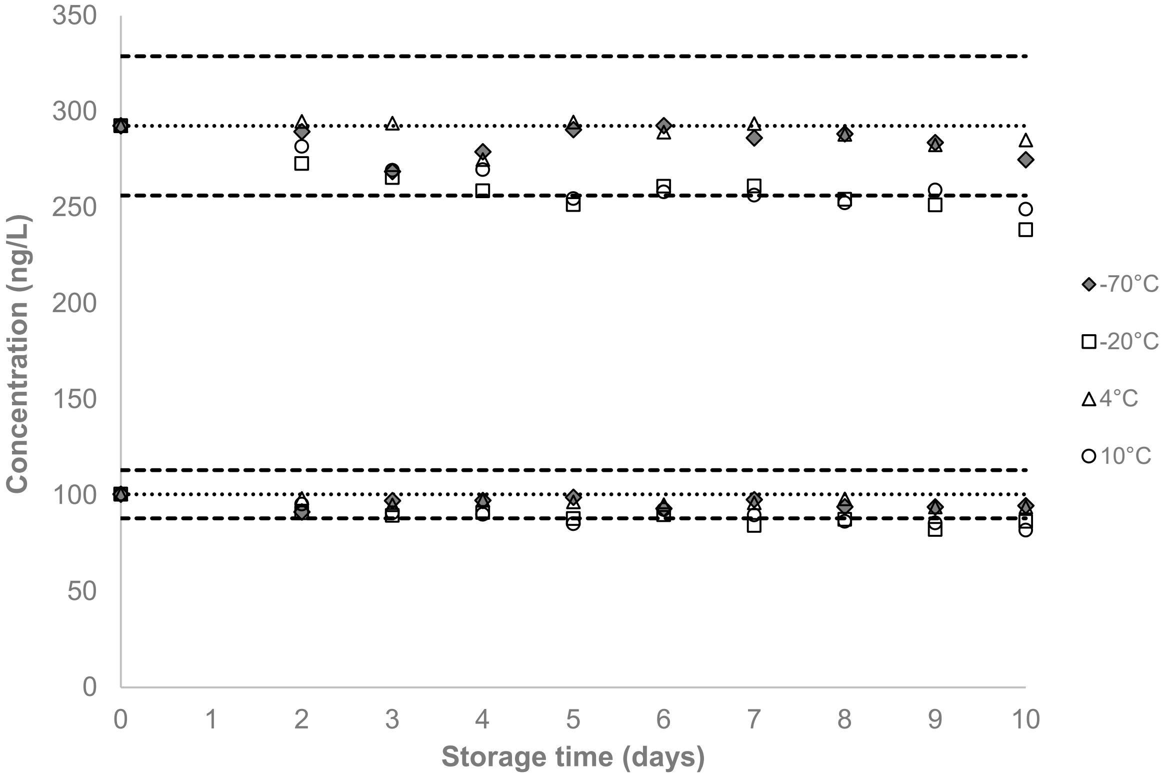

**Dotted line denotes the characterized mean for a given QC pool and corresponding dashed lines denote ±2σ from the characterized mean, as listed in Table 1. The characterized mean for a given QC pool is included as the zero time-point.*

Supplemental Figure S2: Single time-point urinary concentration of 2AMN stored at the different storage conditions for short-term stability testing

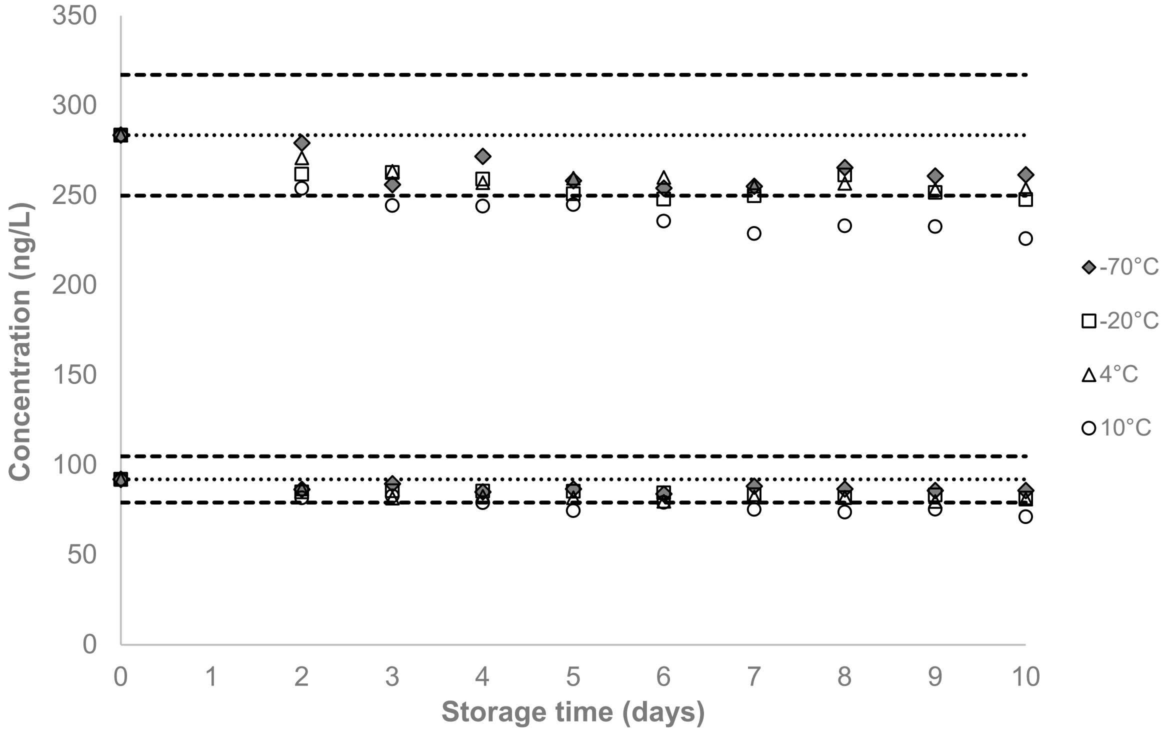

**Dotted line denotes the characterized mean for a given QC pool and corresponding dashed lines denote ±2σ from the characterized mean, as listed in Table 1. The characterized mean for a given QC pool is included as the zero time-point.*

Supplemental Figure S3: Single time-point urinary concentration of 26DM stored at the different storage conditions for short-term stability testing

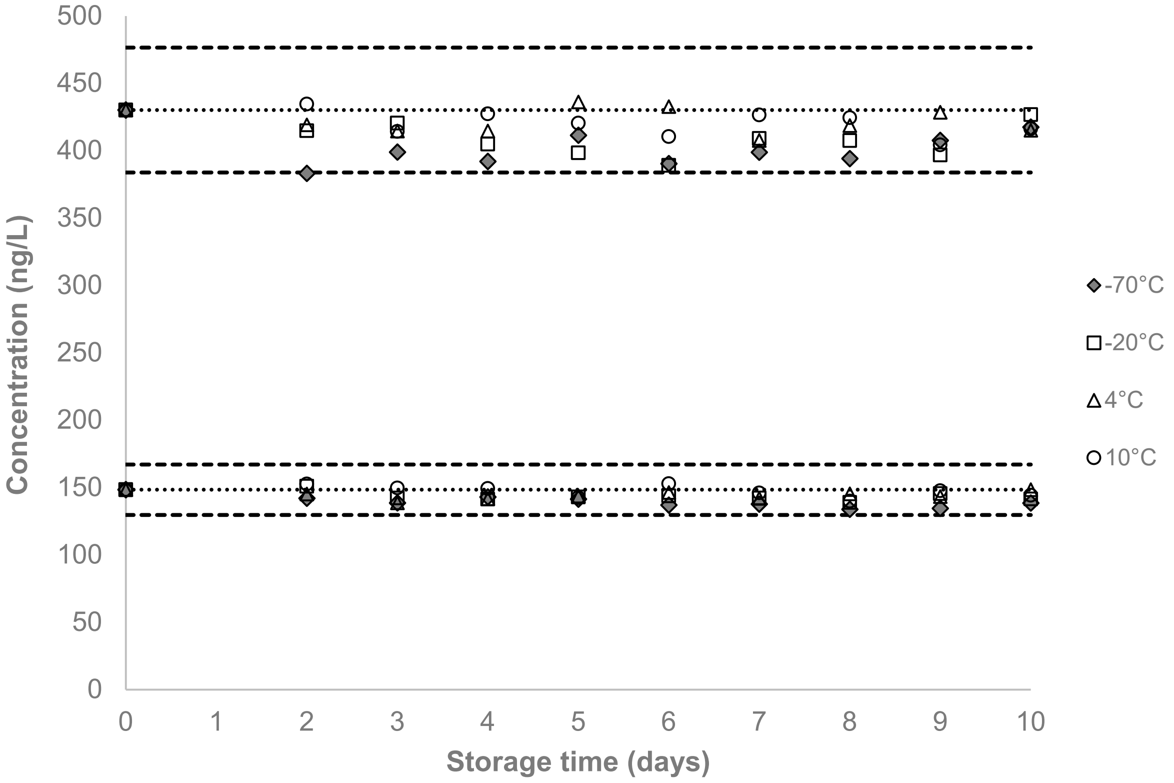

**Dotted line denotes the characterized mean for a given QC pool and corresponding dashed lines denote ±2σ from the characterized mean, as listed in Table 1. The characterized mean for a given QC pool is included as the zero time-point.*

Supplemental Figure S4: Single time-point urinary concentration of OANS stored at the different storage conditions for short-term stability testing

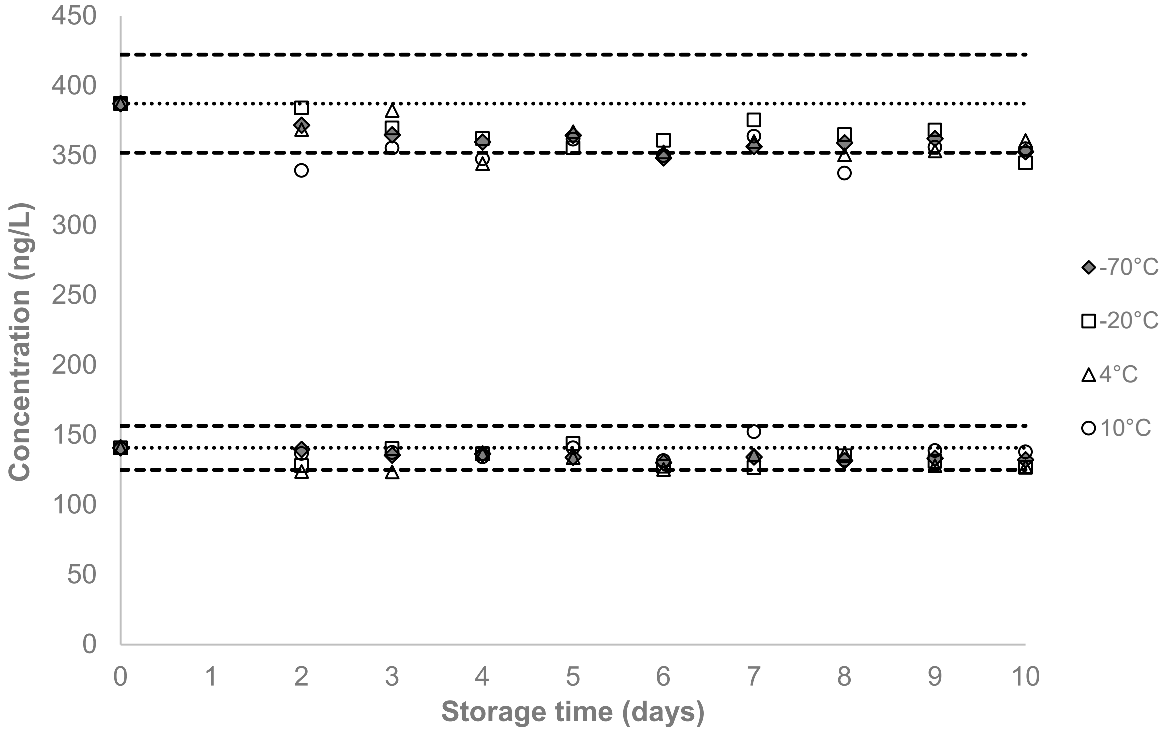

**Dotted line denotes the characterized mean for a given QC pool and corresponding dashed lines denote ±2σ from the characterized mean, as listed in Table 1. The characterized mean for a given QC pool is included as the zero time-point.*

Supplemental Figure S5: Single time-point urinary concentration of OTOL stored at the different storage conditions for short-term stability testing

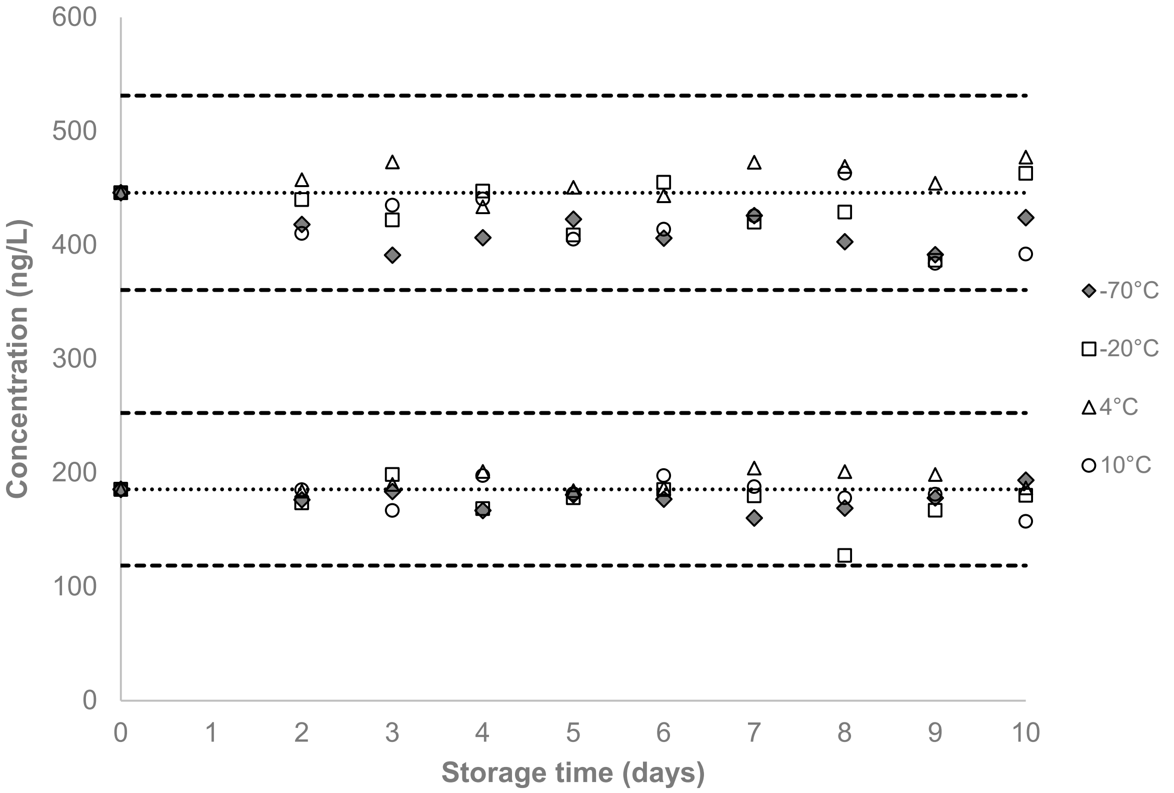

**Dotted line denotes the characterized mean for a given QC pool and corresponding dashed lines denote ±2σ from the characterized mean, as listed in Table 1. The characterized mean for a given QC pool is included as the zero time-point.*

Supplemental Figure S6: Single time-point urinary concentration of 1AMN stored at -70°C for long-term stability testing

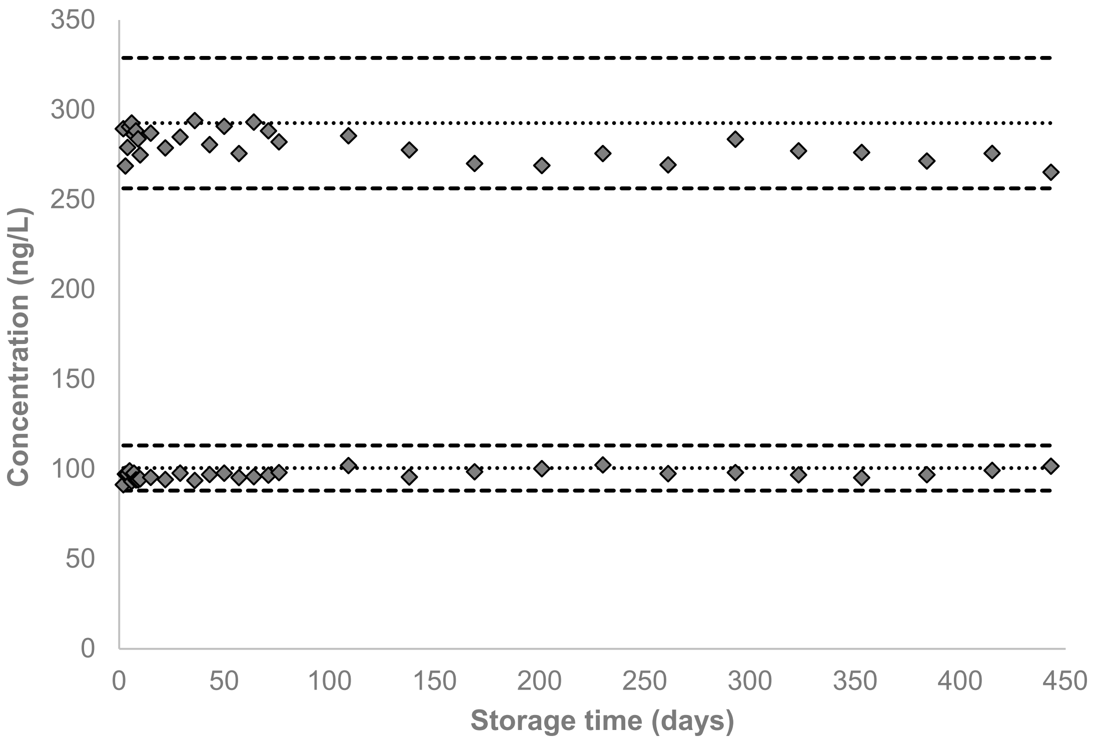

**Dotted line denotes the characterized mean for a given QC pool and corresponding dashed lines denote ±2σ from the characterized mean, as listed in Table 1. The characterized mean for a given QC pool is included as the zero time-point.*

Supplemental Figure S7: Single time-point urinary concentration of 2AMN stored at -70°C for long-term stability testing

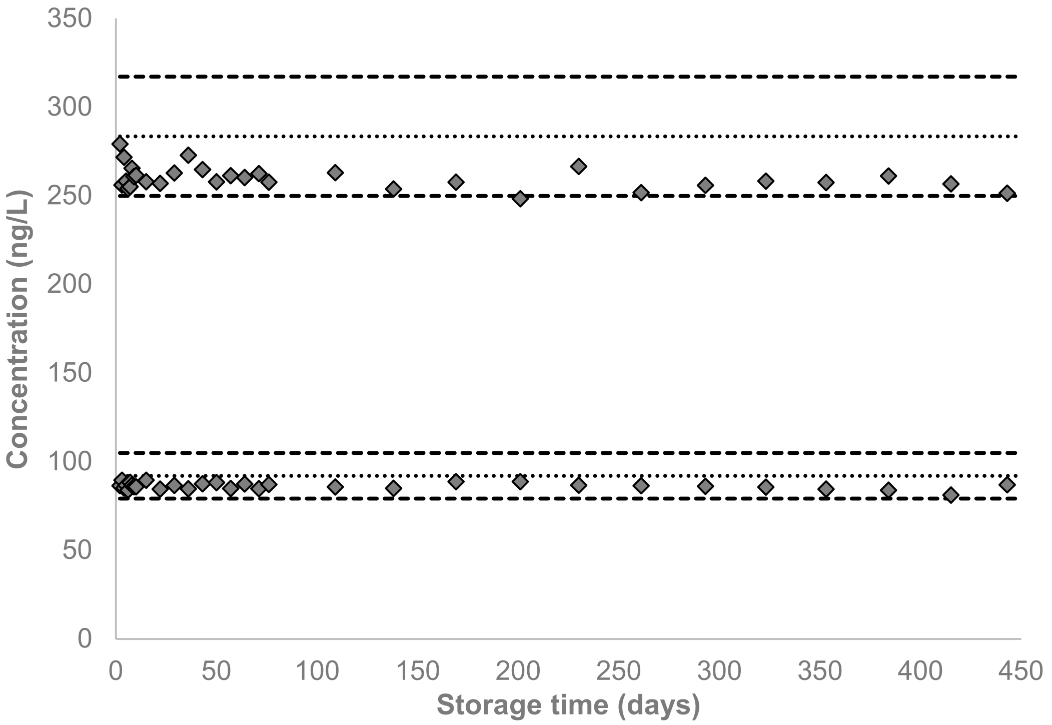

**Dotted line denotes the characterized mean for a given QC pool and corresponding dashed lines denote ±2σ from the characterized mean, as listed in Table 1. The characterized mean for a given QC pool is included as the zero time-point.*

Supplemental Figure S8: Single time-point urinary concentration of 26DM stored at -70°C for long-term stability testing

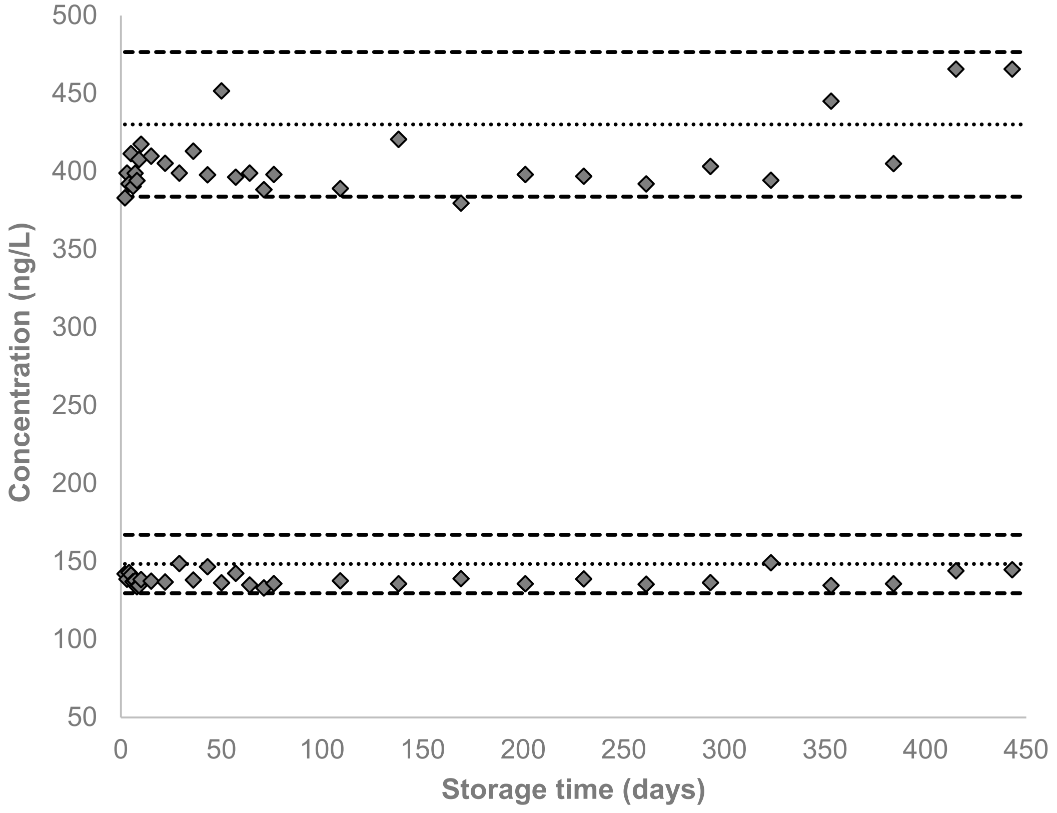

**Dotted line denotes the characterized mean for a given QC pool and corresponding dashed lines denote ±2σ from the characterized mean, as listed in Table 1. The characterized mean for a given QC pool is included as the zero time-point.*

Supplemental Figure S9: Single time-point urinary concentration of OANS stored at -70°C for long-term stability testing

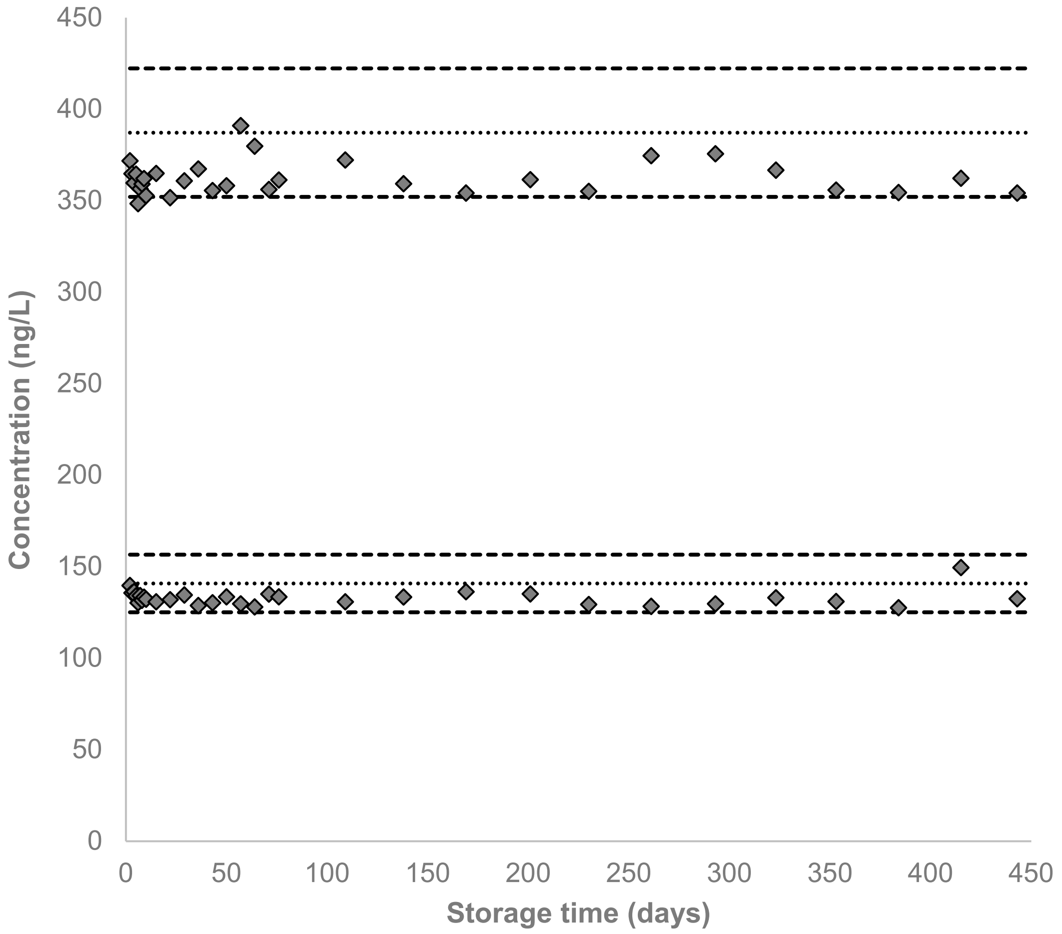

**Dotted line denotes the characterized mean for a given QC pool and corresponding dashed lines denote ±2σ from the characterized mean, as listed in Table 1. The characterized mean for a given QC pool is included as the zero time-point.*

Supplemental Figure S10: Single time-point urinary concentration of OTOL stored at -70°C for long-term stability testing

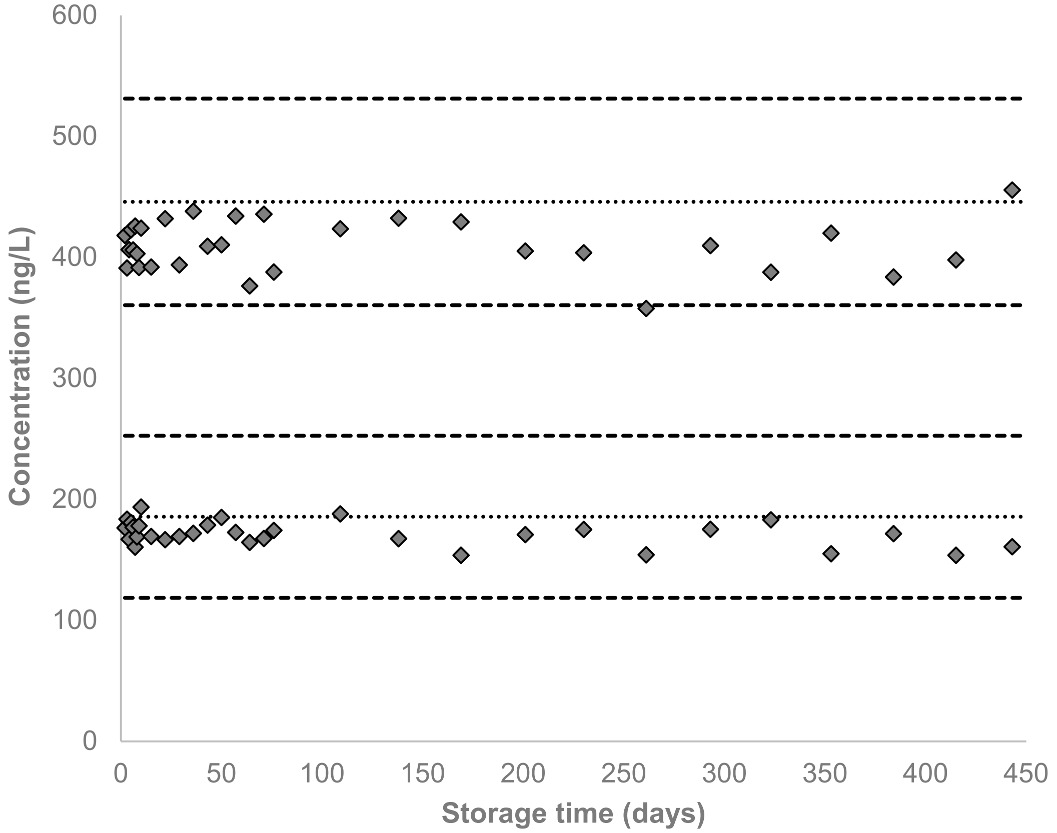

**Dotted line denotes the characterized mean for a given QC pool and corresponding dashed lines denote ±2σ from the characterized mean, as listed in Table 1. The characterized mean for a given QC pool is included as the zero time-point.*

Supplemental Figure S11: Single time-point urinary concentration of 1AMN stored at 20°C for short-term stability testing

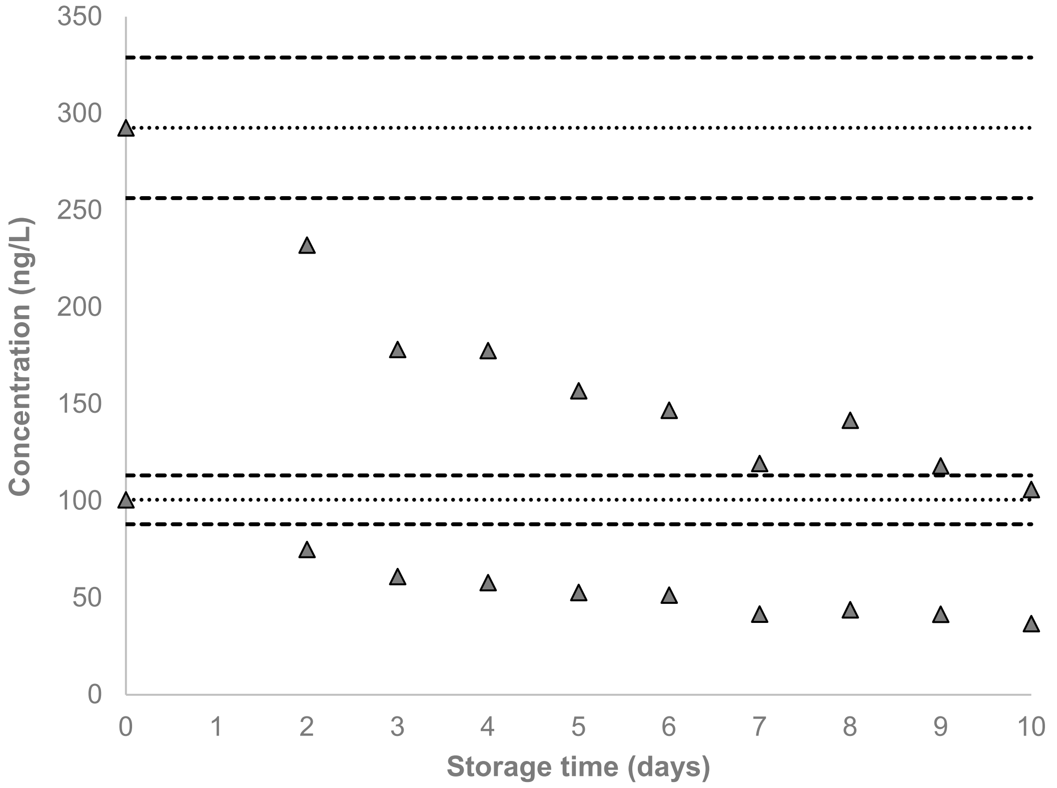

**Dotted line denotes the characterized mean for a given QC pool and corresponding dashed lines denote ±2σ from the characterized mean, as listed in Table 1. The characterized mean for a given QC pool is included as the zero time-point.*

Supplemental Figure S12: Single time-point urinary concentration of 2AMN stored at 20°C for short-term stability testing

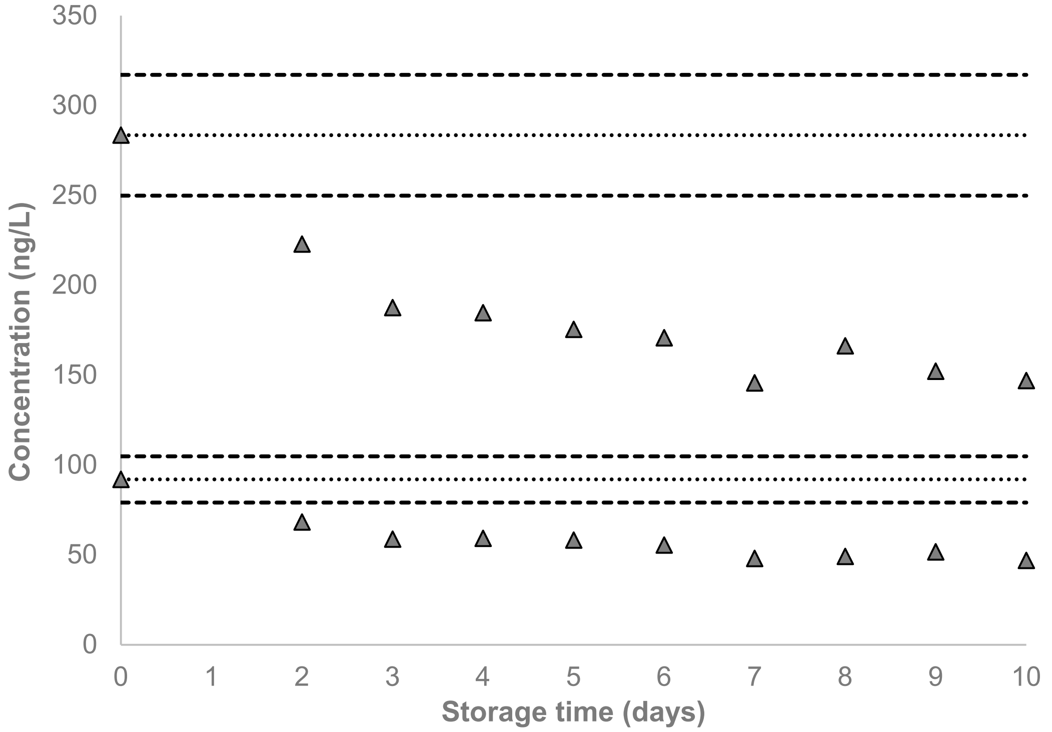

**Dotted line denotes the characterized mean for a given QC pool and corresponding dashed lines denote ±2σ from the characterized mean, as listed in Table 1. The characterized mean for a given QC pool is included as the zero time-point.*

Supplemental Figure S13: Single time-point urinary concentration of 26DM stored at 20°C for short-term stability testing

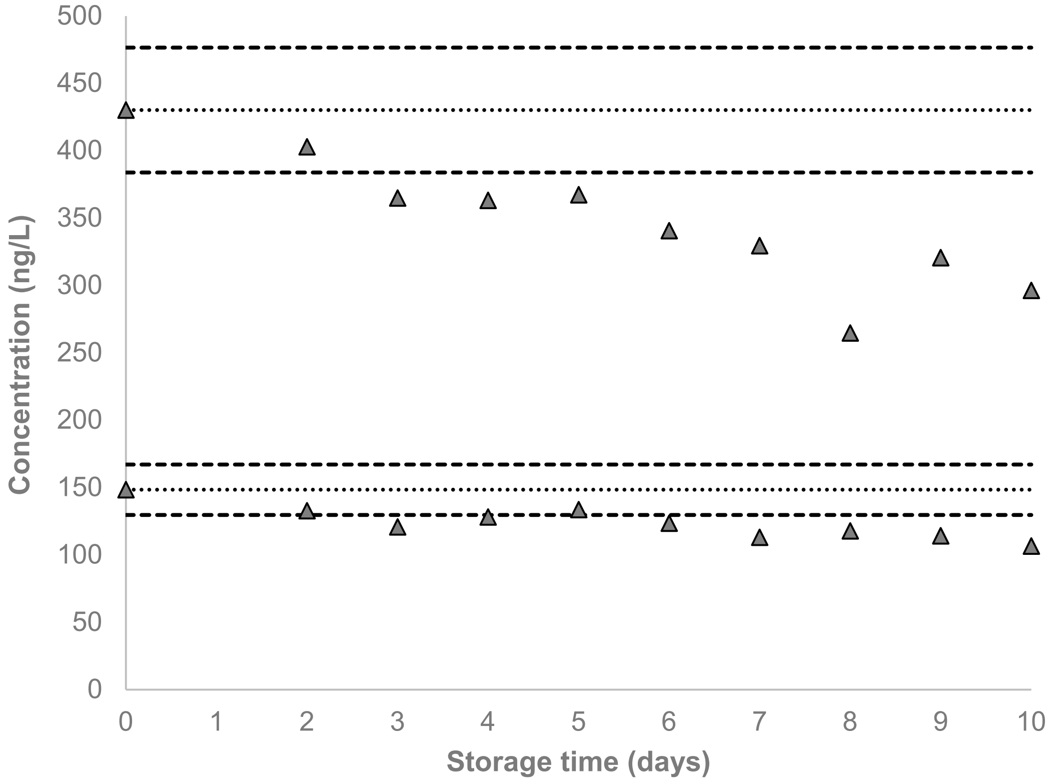

**Dotted line denotes the characterized mean for a given QC pool and corresponding dashed lines denote ±2σ from the characterized mean, as listed in Table 1. The characterized mean for a given QC pool is included as the zero time-point.*

Supplemental Figure S14: Single time-point urinary concentration of OANS stored at 20°C for short-term stability testing

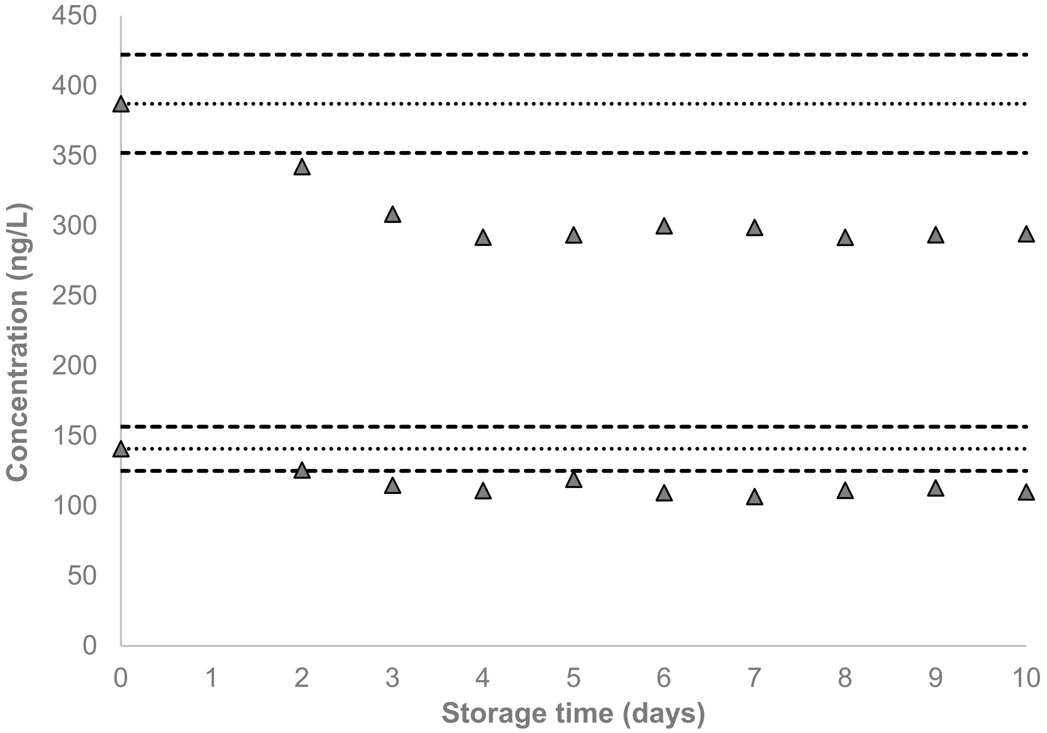

**Dotted line denotes the characterized mean for a given QC pool and corresponding dashed lines denote ±2σ from the characterized mean, as listed in Table 1. The characterized mean for a given QC pool is included as the zero time-point.*

Supplemental Figure S15: Single time-point urinary concentration of OTOL stored at 20°C for short-term stability testing

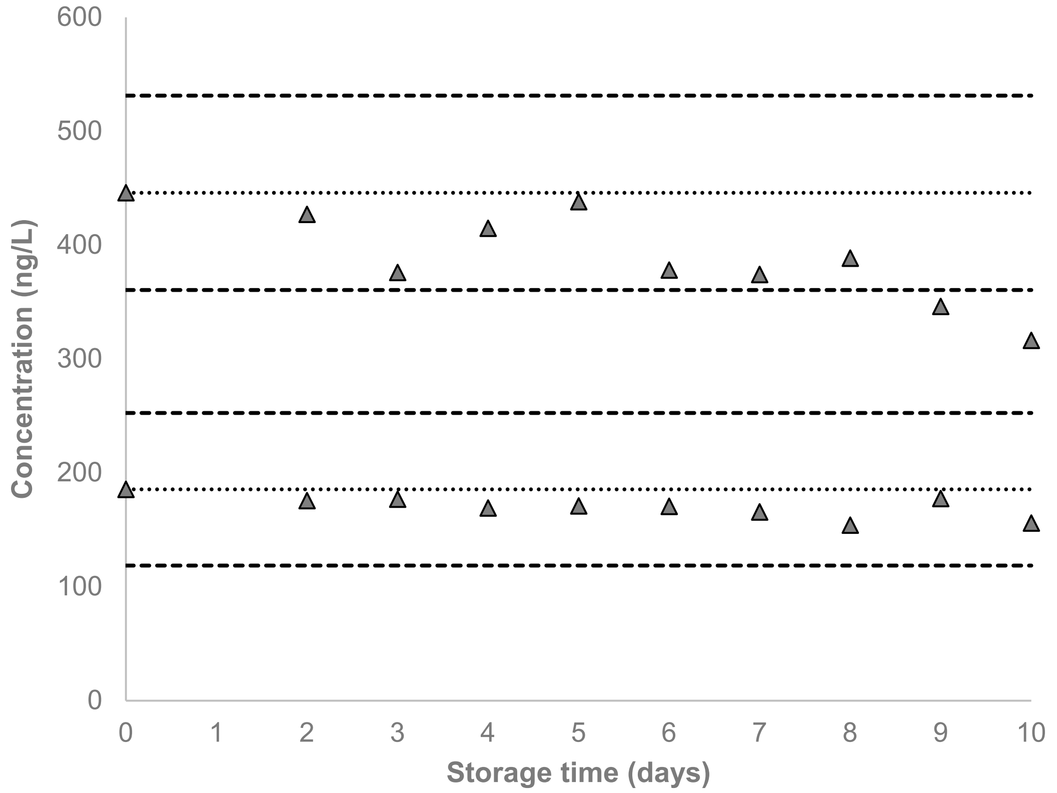

**Dotted line denotes the characterized mean for a given QC pool and corresponding dashed lines denote ±2σ from the characterized mean, as listed in Table 1. The characterized mean for a given QC pool is included as the zero time-point.*

Supplemental Figure S16: The TIC of six quantified AAs and two of the monitored isomers (2ABP and 3ABP) in an urine sample containing approximately 100 ng/L of each target analyte (represented as dashed line) is overlaid on the TIC of a calibration standard containing 100 pg/µL of each target analyte (represented as solid line). The three insets show the related structural isomers of OANS, OTOL and 26DM, each spiked at 100 pg/µL.

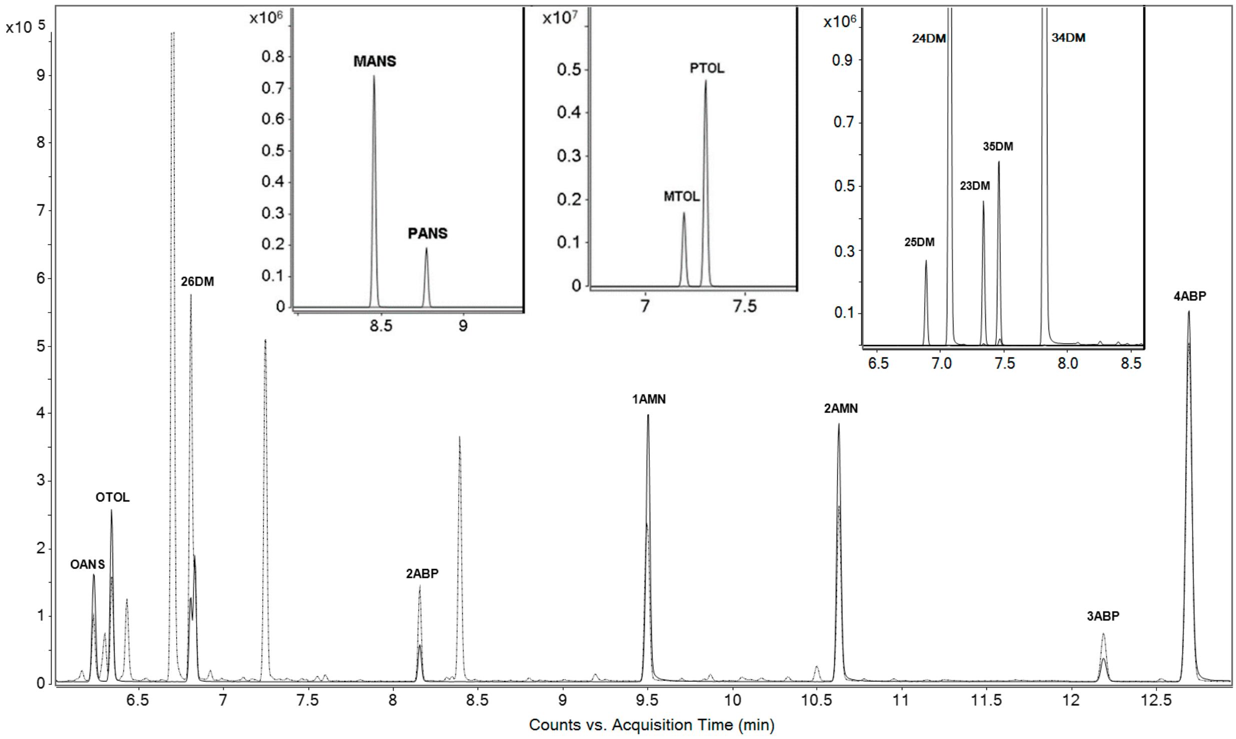

Supplement: Supplementary file 1 [file ijerph-20-04135-s001.zip › ijerph-2190623-supplementary.pdf]
